# Interaction patterns of systemic problems in distributed energy technology diffusion: a case study of photovoltaics in the Western Cape province of South Africa

**DOI:** 10.1080/09537325.2018.1473851

**Published:** 2018-05-15

**Authors:** Michael Kriechbaum, Alan C. Brent, Alfred Posch

**Affiliations:** a Institute for Systems Sciences, Innovation & Sustainability Research, University of Graz, Graz, Austria; b Department of Industrial Engineering, and the Centre for Renewable and Sustainable Energy Studies, Stellenbosch University, Stellenbosch, South Africa; c Sustainable Energy Systems, Engineering and Computer Science, Victoria University of Wellington, Wellington, New Zealand

**Keywords:** Technological innovation systems, systemic problems, technology diffusion, photovoltaics

## Abstract

Compared to large-scale renewable energy systems, distributed systems have diffused relatively slowly in recent years, particularly in developing countries. In this study, we analysed the barriers to the diffusion of distributed photovoltaics in South Africa by applying the technological innovation system framework. More specifically, we carried out an interview-based structural-functional analysis to identify underlying systemic problems, reveal their patterns of interaction, and analyse the implications of these patterns for the relationship among the key processes within the innovation system, the so-called system functions. We identified a variety of interlinked problems, shed light on the specific roles of different, mal-performing, system functions, and revealed constellations where specific functions blocked each other and, thereby, created lock-in situations. Although the findings reveal the complexity of the problems that are associated with the implementation of distributed energy technologies, they also indicate that these problems may be addressed successfully.

## 1. Introduction

The decarbonisation of our society is one of the main challenges of the 21st century. The energy sector will play a key role in this regard (Grin, Rotmans, and Schot 2011). To radically reduce fossil fuel consumption, countries all over the world will have to increase the use of renewable energy technologies (IEA and IRENA 2017). In this transition process, distributed types of renewable energy production and, in particular, distributed solar photovoltaic (PV) technologies (i.e. residential, commercial and industrial applications) may play an important role as well (IEA and IRENA 2017). Thus far, however, distributed photovoltaics accounted for a relatively small proportion of the rising renewable energy production, whereas their share was particularly low in developing countries (FS-UNEP 2016). South Africa gives a good example in this respect. It's rapidly growing renewable energy market, which has become one of the major markets in the global South, was mainly driven by large, utility-scale systems, whereas distributed PV systems lagged significantly behind (PQRS 2016).

Whereas standard economic theories of innovation associate barriers to technological change primarily with mechanisms of market prices, a growing number of scholars have started to turn their attention towards the socio-institutional structures that underlie innovation processes. One of the conceptual frameworks that has arisen from these efforts is the technological innovation system (TIS) framework. This framework describes the networks of actors and institutions (i.e. the socio-institutional system) that emerge behind technologies and suggests that barriers to the development and diffusion of novel technologies are typically rooted in weaknesses within these networks (Markard, Raven, and Truffer 2012). By adopting a systemic view on innovation problems, the TIS literature contributed to the development of a new rationale for policy intervention that shifts the focus away from market failures and towards system failures or systemic problems (Smith 2000; Weber and Rohracher 2012; Bleda and Del Río 2013).

In order to identify systemic problems, TIS scholars traditionally have dealt either with the structure of the innovation system by focussing on the malfunctioning or absence of specific system components (e.g. Klein Woolthuis, Lankhuizen, and Gilsing 2005; Negro, Alkemade, and Hekkert 2012), or with the weaknesses of the key processes that take place within innovation systems, the so-called system functions (e.g. Negro and Hekkert 2008; Praetorius et al. 2010). Recently, Turner et al. (2016) and Wesseling and Van der Vooren (2016) combined both structural and functional perspectives, and conceptualised systemic problems at the interface between functional weaknesses and structural deficiencies. Following earlier work on system functions that analysed how weak functions interrelate and create patterns of cumulative causation by reinforcing each other (see e.g. Hillman et al. 2008; Suurs et al. 2010), they studied how these reconceptualised systemic problems interact with each other. However, although these scholars revealed interesting dynamics, and even identified self-reinforcing tendencies, they only scarcely dealt with the dynamics among the system functions and, thus, with earlier work on cumulative causation dynamics.

In this paper, we connected the dynamics among systemic problems to those among system functions more explicitly by applying an interview-based structural-functional analysis of distributed PV technology in the Western Cape Province of South Africa. Thereby, the paper not only demonstrates the usefulness of the TIS framework, but also sheds light on problem structures and systemic lock-in situations that may arise with regard to the implementation of distributed renewable energy technologies. The remainder of the paper is structured as follows: in the next section, the current literature on the TIS framework, its functions approach, and the associated concept of systemic problems is reviewed. In the third section, the methodological approach of the analysis is explained. Subsequently, the identified systemic problems and their interaction patterns are demonstrated, and it is shown how these interaction patterns related to the dynamics among system functions. In the last two sections, the findings are discussed and conclusions are drawn.

## 2. The TIS Framework & the notion of systemic problems

The concept of technological innovation systems (TIS) emerged as part of the broader literature on innovation systems (Markard and Truffer 2008). Technological innovation systems are regarded as ‘networks of agents interacting in a specific economic area under a particular institutional infrastructure and are involved in the generation, diffusion and utilisation of technology’ (Carlsson and Stankiewicz 1991, 111). The TIS framework is particularly useful for identifying barriers to innovation. Based on empirical studies, TIS scholars have identified numerous systemic factors or systemic problems that can hamper innovation processes (Negro, Alkemade, and Hekkert 2012). Most of the early literature on systemic problems focuses on structural deficiencies. In other words, it identifies missing or malfunctioning parts of the structure of a technological innovation system. Klein Woolthuis, Lankhuizen, and Gilsing (2005), for instance, conceptualised four main categories of systemic problems in terms of their related system components (see Table 1).

**Table 1.** Conceptualisation of systemic problems as structural deficiencies (based on Klein Woolthuis, Lankhuizen, and Gilsing 2005).

Systemic Problems	Explanation
*Infrastructural Problems (physical & knowledge)*	Infrastructure (physical, knowledge, financial infrastructure) is absent, inadequate or malfunctioning.
*Institutional Problems (hard & soft)*	Specific (soft and/or hard) institutions are absent, too stringent (appropriability trap) or too weak, or malfunctioning.
*Interaction Problems too strong & too weak)*	Networks are missing (e.g. cognitive distance between actors, lack of trust), too strong (e.g. myopia, no ‘windows of opportunities’), or too weak (weak connectivity of actors).
*Capabilities Problems*	Relevant actors may be absent or lack competence, learning capacities, skills for developing visions & strategies, etc.

While the early literature focuses on structural deficiencies, more recent work highlights so-called functional weaknesses. The notion of functional weaknesses is based on the idea that each innovation system needs to fulfil certain system functions in order to develop and operate successfully. While the overall goal of technological innovation systems is to generate, diffuse and utilise technology, system functions represent the tasks that are actually required in order to achieve this goal (e.g. Hekkert et al. 2007). According to Jacobsson and Bergek (2011, 46), system functions are the ‘intermediate variables between structure and performance’. Table 2 shows the most common categorisation of system functions. One of the main benefits of the functions approach is that it allows scholars to better measure the performance of innovation systems. Furthermore, it highlights the actual weaknesses that policy-makers need to tackle: while a structural imperfection (e.g. the absence of a certain actor) might not always constitute a problem, a poorly performing system function more clearly indicates the need for policy intervention (Bergek et al. 2008).

**Table 2.** Set of system functions (based on Hekkert et al. 2007; Bergek et al. 2008).

System Functions	Explanation
*Creation of Legitimacy*	Refers to all conscious lobbying actions that help to increase the acceptance of innovations.
*Knowledge Development*	Based on two key activities; namely ‘learning by doing’ and ‘learning by searching’. There are different types of knowledge (e.g. scientific knowledge, technological knowledge etc.) as well as different sources of knowledge (e.g. R&D, pilots).
*Knowledge Diffusion*	Happens mainly through network activities and is crucial for the decisions that are being made by heterogeneous innovation actors, including research, government and competitors.
*Market Formation*	Refers to all activities that help to overcome difficulties to enter the market (e.g. poor performances, inefficiencies, lock-in and path dependencies created within established sectors etc.).
*Resource Mobilisation*	Describes all processes that are carried out in order to access and secure resources (competences/human capital, financial capital, complementary assets).
*Guidance of the Search*	Refers to all activities that help innovations to ‘survive’ the selection environment (e.g. by raising expectations).
*Entrepreneurial Activity*	Highlights the importance of entrepreneurs in turning the ‘potential of new knowledge development, networks and markets into concrete action to generate and take advantage of business opportunities’ (Hekkert et al. 2007, 421).

Another benefit of the functions approach is that it provides scholars with certain insights into the growth processes of innovation systems. Owing to their complex structures, innovation systems exhibit dynamics that are characterised by non-linearity, reciprocity and feedback loops (Hillman et al. 2008). According to several scholars, interaction patterns among system functions well illustrate such dynamics (e.g. Suurs and Hekkert 2009; Suurs et al. 2010; Bergek et al. 2008). More specifically, they showed that individual system functions may strongly interact with each other and in some cases even may create patterns of cumulative causations,^1^ where interacting functions induce reinforcing or antagonistic feedback loops. Such cumulative causation patterns may explain both rapid take-offs as well as breakdowns of innovation systems. Building on this notion, Suurs and Hekkert (2009), for instance, introduce the concept of so-called ‘motors of innovation’, as reinforcing feedback loops that cause formative innovation systems to grow endogenously.

Functional dynamics are, however, always dependent on the system structure. Bergek et al. (2008), for instance, regard functional analysis as being a complementary step that scholars should undertake in addition to structural analysis. In fact, they suggest applying the functions approach in order to identify those parts of the system structure that are in greatest need of policy intervention. Suurs (2009) perhaps best described the relationship between structure and functions, when he compared structure and functions to ‘two different sides of the same coin’. Similarly, Wieczorek and Hekkert (2012) acknowledge that ‘the reason why a certain system function is absent or weak can be related to the structure of the innovation system’. Based on this assumption, they propose identifying systemic problems by applying a coupled functional-structural analysis, according to which each system function is analysed with regard to structural deficiencies; in other words, they conceptualised systemic problems as lying at the interface between functional weaknesses and structural deficiencies.

Accordingly, the analysis of systemic problems provides useful insight into the nature of mal-performing system functions. Moreover, systemic problems may well explain why and how weak system functions interrelate or even reinforce each other. However, scholars only recently started to empirically map the relationship between systemic problems and system functions. Whereas Turner et al. (2016) showed how New Zealand's agricultural sector (and its ability to innovate) suffered from interdependent systemic problems that caused system functions to perform weak, Wesseling and Van der Vooren (2016) demonstrated functional weaknesses and their underlying systemic problems in the context of the decarbonisation of the Dutch concrete sector. In both of the studies, the scholars identified systemic lock-in situations, where systemic problems reinforced each other and fuelled a vicious cycle that blocked the overall performance of the innovation system. However, relatively little attention was paid to the question of how the identified patterns related to the dynamics among system functions, and to their respective roles within the innovation system.

## 3. Conceptual model of the analysis

**Figure 1.**
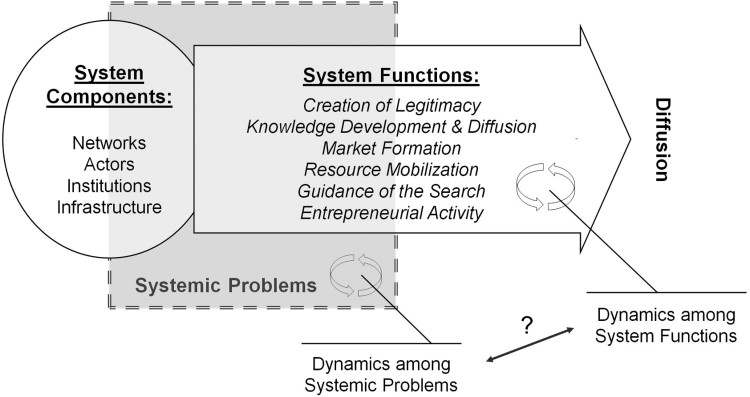
The conceptual model of our analysis (based on Hekkert et al. 2007; Bergek et al. 2008; Wieczorek and Hekkert 2012).

In the conceptual model of our analysis, systemic problems were defined as all factors that hinder the innovation system from operating and carrying out its system functions. In addition, we conceptualised systemic problems as being those lying at the interface between functional weaknesses and structural deficiencies in order to acknowledge the strong interdependencies between mal-performing system functions and the absence or malfunctioning of specific components of the innovation system (Wieczorek and Hekkert 2012). The system components were classified according to Klein Woolthuis, Lankhuizen, and Gilsing 2005. The key emphasis of our analysis was on the question of how dynamics among systemic problems relate to the dynamics among system functions, and the role of the specific functions within the innovation system (see Figure 1).

The system functions were classified in accordance with the scheme outlined by Hekkert et al. (2007),^2^ except that we integrated knowledge development and knowledge diffusion into one function (Bergek et al. 2008). Furthermore, we refined the concept of knowledge in order to make the function more applicable to adopting regions or, more precisely, to questions relating to the diffusion of a relatively mature innovation. While TIS scholars usually limit the notion of knowledge to the information industry requires for generating innovative products or services, we also included knowledge on how to implement the respective technology and knowledge of potential technology users about the technology. This is in line with the notion of absorptive capacity, which Blum, Bening, and Schmidt (2015) introduced in order to account for the ability to utilise new technologies and adapt them to the local context.

## 4. Methods

To gain an in-depth insight into the nature and the dynamics of systemic problems that were associated with distributed PV technology in South Africa, we selected the Western Cape Province as a case study and employed a qualitative research design based on semi-structured expert interviews, which we conducted in 2013; the year that the PV industry started gaining traction in the country, and the province. Like in the rest of the country, the Western Cape's rising renewable energy market had been mainly driven by large-scale facilities at that time, while distributed PV systems (i.e. residential, industrial and commercial installations) played an insignificant role (Rix et al. 2015). The reason for why we selected the Western Cape Province was that despite the low diffusion rate, it took a leading role by showing beginning efforts to support the technology (e.g. by implementing pilot projects of grid-connected systems).

There were three main reasons for adopting a qualitative case study design based on interviews. First, we wanted to carry out an explanatory analysis and produce new conceptual as well as empirical insights with respect to the interaction patterns of systemic problems. Second, we regard the barriers to technology diffusion as a contemporary phenomenon that needs to be studied within its real-life context. This is in contrast to the method of history event analyses, which draws on historic-longitudinal data and is commonly applied in the TIS literature.^3^ Third, due to the lack of data, with an industry in its infancy, it was not possible to quantitatively describe the key variables of our conceptual framework.

In total, we interviewed 14 experts, whom we identified and recruited via online research, media reports, event visits (e.g. conferences, workshops), and the method of snowball sampling (recommendations of other interviewees). Based on the notion of experts developed by Meuser and Nagel (1991), we defined experts as persons who (i) have privileged access to information about the regional solar industry and associated actor groups, and/or (ii) are responsible for the development, implementation or control of relevant strategies or policies. Furthermore, we intended to reach a broad spectrum of society in our sample and included representatives of academia, government, business and civil society (see Table 3). Again, with the industry being at its infancy in 2013, only a small number of experts were active in the distributed PV industry in the Western Cape Province, and thus the relatively small sample of 14 is deemed representative.The recorded interview sessions were organised as follows: each interviewee received a broad description of the research in advance so that he/she was able to prepare for the interview. We divided the interview sessions, which took 1,5–3 h, into two parts. First, open questions were asked in order to enable the interviewee to freely give his/her opinion on the topic – a typical first question, for example, was: ‘What is the current situation with respect to distributed PV technology in the Western Cape Province?’ Second, during the course of the interview, the questions became more specific. For this an individual list of questions were developed and tailored to the expert's specific field of expertise. For instance, interviewees from the private sector (i.e. installers and consultants) were specifically asked to describe (i) the current entrepreneurial landscape that has emerged around distributed PV technology, (ii) the related challenges and barriers, as well as the (iii) past developments and future outlooks.

**Table 3.** Overview of interviewees.

Group of Society	Code	Person	Position of Interviewees
Academia	ACD	1	Senior Researcher, head of research group on renewables
		2	Researcher, specialised on the local diffusion of renewables
		3	Senior Researcher, involved in multiple research projects on solar photovoltaics
Public sector	PBS	1	Renewable energy manager (Province)
		2	Principal engineer (Municipality)
		3	Project manager renewable energy (Province & Municipality)
Non-governmental organisations	NGO	1	Programme manager, Project 90 Cut Carbon
		2	NGO member, WESSA
		3	NGO member, Earth Life Africa
Private sector (companies)	PRV	1	Energy consultant (focus on renewable energy)
		2	PV installer (pioneering company)
		3	PV installer (start-up company)
		4	Energy consultant (involved in several research projects on solar PV)
End-user	EDU	1	Pioneer and operator of the first grid-connected residential system in the Province

**Figure 2.**
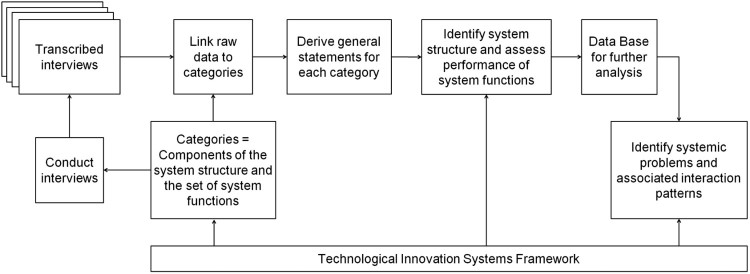
Data Processing Steps (adapted from Gläser and Laudel 2013).

After the interviews were transcribed and stored in an adequate database, we conducted a qualitative content analysis (Duriau, Reger, and Pfarrer 2007; Mayring 2000) in order to identify systemic problems and the associated patterns of interaction. Because we took a deductive approach, which is typically used for ‘validating or extending conceptually a theoretical framework or theory’ and is particularly helpful for determining the relationships among the variables of interest derived from the literature (Hsieh and Shannon 2005, 1281), our conceptual framework served as the basis for both the framework of topics that was to be explored in the interviews and the subsequent analysis. Figure 2 shows how the theoretical framework related to the analysis and gives a detailed illustration of the data processing steps. To minimise the risk of analytical biases, which particularly scholars that adopt theory-driven research designs are exposed to, we applied the methodological procedures of (i) making constant comparisons, (ii) considering negative cases, and (iii) employing rival thinking (Yin 2014). Furthermore, the information received was triangulated, as far as possible, by including data from the secondary literature, i.e. from relevant reports, newspaper articles, legal documents and homepages of important actors. For a better traceability of the analysis, the specific society group of the interviewees are referenced in the findings section by using the abbreviation code demonstrated in Table 3.

## 5. Findings

In this section, the identified systemic problems (i.e. the factors that hindered the performance of the system functions) are described and their linkage to the system structure is revealed. Then the interaction patterns among systemic problems are shown and the implications of these patterns for the relationship among system functions and for their specific roles within the innovation system are demonstrated.

### 5.1. Systemic problems

Each of the system functions suffered from several systemic problems that effectively blocked them from operating.

**Figure 3.**
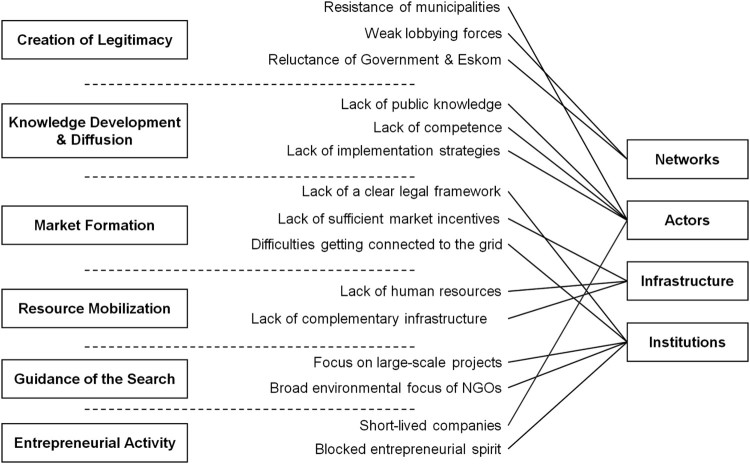
Systemic problems and their linkages to the system functions and to the system structure.

#### 5.1.1. Creation of legitimacy

The most severe problem within the creation of legitimacy related to the resistance of the municipalities, which the interviewees unanimously made a subject of discussion (ACD, NGO, PRV, EDU). The municipalities were strongly involved in the power sector and generated income through buying electricity from Eskom, the state-owned energy provider,^4^ and through selling that electricity at a marked-up price to the end consumers (residential households, industries and commercial businesses). According to the interviewees, the municipalities resisted against distributed photovoltaics, because they feared that a wide utilisation of this technology would lower the electricity demand of the end consumers and, eventually, the municipalities’ income. When asked about this fear, the interviewed officials agreed, but also justified the fact of profit generation by asserting that such income was largely used for cross subsidising poor households. One of the officials, for instance, said that they ‘would like to support PV technology, but only if it is not at the expense of poor communities’. With regard to the creation of legitimacy, the interviewees further highlighted the reluctance of Eskom and governmental authorities, which did not seem to oppose the technology, but also did not support it (PRV, PBS, EDU, ACD). For instance, responsibilities with respect to the grid-integration of distributed PV systems were completely delegated to the municipalities. Finally, the interviewees pointed at the absence of strong initiatives or groups that successfully lobbied for the technology (NGO, EDU).

#### 5.1.2. Knowledge development & diffusion

One of the main problems mentioned with regard to the *development & diffusion of knowledge* related to the perceived lack of an understanding how to implement distributed PV systems. The interviewed officials, in particular, pointed out that it was not clear how to integrate distributed PV technology in the overall electricity grid. The municipalities were lacking knowledge concerning how exactly the technology would affect municipalities financially, and did not have strategies that allow them to connect PV systems to the grid while maintaining or optimising their revenue (PBS, ACD). Another problem, which was revealed within this function, relates to the lack of public knowledge about the technology (RES, NGO, PRV). Related to this, the interviewees mentioned that large parts of the population (particularly of poorer communities) were not even aware of the existence of the technology (NGO, PRV). Furthermore, the lack of sufficient competence of firms (e.g. lack of technical knowledge or insufficiently qualified workforces) trying to enter the residential PV market was mentioned (PRV, PBS).

#### 5.1.3. Market formation

Three main barriers to the market formation of distributed PV technology were revealed. The first barrier relates to the perceived difficulties with connecting to the grid. Unanimously the interviewees told that it was almost impossible to get permission from municipalities^5^ for grid-connected systems, and that without this it was impossible to run a PV system cost-efficiently. Further, the interviewees highlighted the lack of a clear legal framework for the utilisation of PV technology (PBS, PRV, ENU). They predominantly criticised the energy regulator NERSA, which had determined that the distributors (hence in most cases the municipalities) needed to regulate and integrate small-scale and medium-scale generators, but had refrained from designing a framework for how this should be done. Furthermore, interviewees pointed to the fact that the success of distributed PV technology strongly depended on financial incentive schemes, which were missing, particularly in the residential sector (PRV).

#### 5.1.4. Resource mobilisation

The interviewees revealed weaknesses with regard to mobilising human resources and complementary assets or infrastructure (ACD, PRV, PBS). One of the representatives of academia, for instance, highlighted, that her research centre was the only one of two in the region that focused on renewable energy, and that it counted less than 30 people, and thus was not in a position to meet the actual demand for research. One of the installers cited another example, which pointed at the municipalities’ lack of suitably qualified administrative staff when dealing with PV-related requests: ‘I have spent hours on the phone trying to find out what I should do to actually get approval. And anybody could help me.’ With regard to the lack of complementary infrastructure, the interviewees mentioned severe problems related to the availability of an appropriate technological infrastructure, such as smart metering devices and associated software (PBS, PRV, ENU).

#### 5.1.5. Guidance of the search

The interviewees mentioned two main issues that related to the function guidance of the search. First, they reported that both in the public discourse on renewables and in the renewable energy programmes of Eskom, policy makers and universities, small-scale systems are barely considered (PBS, ACD). Indeed, we found only one research report that dealt with distributed PV systems and associated benefits (e.g. Urban-Econ et al. 2013), and the available energy policy documents revealed that regional policy initiatives largely ignored distributed types of energy production (e.g. GreenCape 2014; CoCT 2014). Second, the interviewees mentioned that NGOs, which could make the technology more visible among society, usually had a very broad environmental programme that focussed on general topics (e.g. conservation, climate change, energy use) and paid only little attention to specific technologies such as distributed PV (NGO).

#### 5.1.6. Entrepreneurial activity

The distributed PV sector was described as short-lived entrepreneurial landscape, one that is characterised by the hope and frustration at the same time. According to the interviewees, small firms were not able to stay in the distributed, and in particularly in the residential, market for long (PBS, PRV). They explained this by pointing to the identified lack of competence among such firms and to the fact that the core business of many small installers was the Solar Water Heater market; i.e. such businesses installed PV systems only occasionally, and when there was enough demand (PRV, PBS). In essence, it was indicated that businesses did recognise the potential of the market but could not turn this potential into profit (PRV, PBS, NGO, ENU).

### 5.2. Systemic problems at the structural-functional interface

The systemic problems identified not only reveal weaknesses of the system function, but also what parts of the system structure were affected (Figure 3). In other words, it was possible to trace each of the systemic problems back to the absence or weakness of one of the structural components. For instance, both the lack of a clear legal framework and the focus on large-scale projects constituted to an absence of required (formal and informal) institutions. The lack of human resources gives an example of an infrastructural problem, because it related to the lack in qualified staff in administration and research and thus to a missing or malfunctioning knowledge infrastructure. As another example, the reluctance of the governmental authorities and the absence of strong lobbying forces were network-related problems. While the governmental authorities (such as ministries, regulatory agencies or utilities) seemed to constitute a network that is too strong and hardly willing to interact with actors dealing with distributed PV technology, lobbying networks that supported PV technology were far too weak.

### 5.3. Patterns of interaction

**Figure 4.**
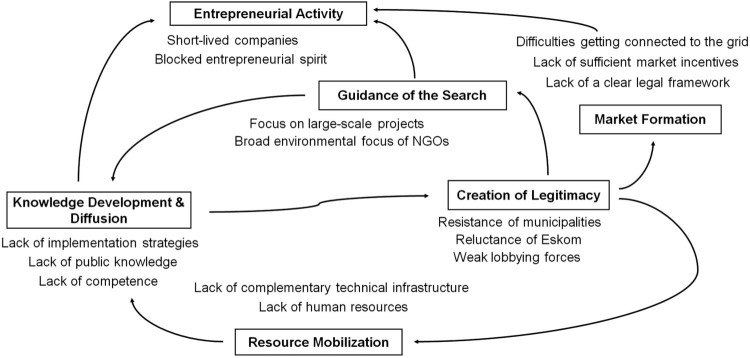
Relationship between system functions. Each of the arrows represent a situation where problems within one function were contributing to problems within another function.

**Figure 5.**
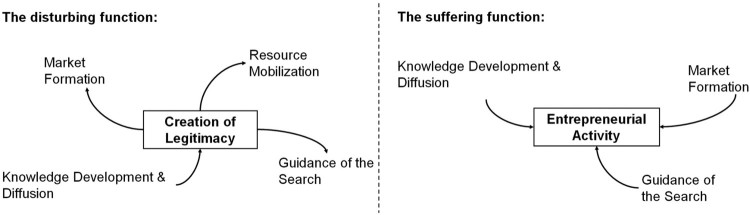
While the weak function creation of legitimacy was contributing to problems of several other functions, the weak function entrepreneurial activity was affected by problems of several other functions.

The analysis shows that the identified systemic problems strongly interacted with each other. Furthermore, these interaction patterns reveal how the corresponding system functions interrelated or, more precisely, how problems within one function related to problems within other functions (Figure 4). Related to this, the patterns also show that different functions played different roles within the innovation system. The two functions *creation of legitimacy* and *entrepreneurial activity* best illustrate these different roles. Problems within *creation of legitimacy* had a wide impact across the innovation system and caused or at least contributed to problems within three other functions. By contrast, the problems within *entrepreneurial activity* did not hamper the performance of any other function, but were negatively affected by problems within three other functions instead. This is why we called *creation of legitimacy* the ‘disturbing’ function and *entrepreneurial activity* the ‘suffering’ function (Figure 5). *Knowledge development & diffusion* played a particularly interesting role as well. It was the only function whose problems contributed to the weak performance of the ‘disturbing’ function *creation of legitimacy* and, together with the *creation of legitimacy*, it had the highest number of linkages with other functions across the system.

#### 5.3.1. Creation of legitimacy – the disturbing function

Problems within creation of legitimacy contributed to problems within three other functions and thus had a wide impact across the innovation system. First, there was a clear link to the problems of the function *market formation*. Because of their negative stance towards distributed photovoltaics, municipalities did not allow PV operators to connect to the grid and had also no interest to implement effective market incentives such as feed-in tariffs (PRV, PBS, NGO, ENU, ACD). Furthermore, the interviewees traced the lack of a clear legal framework to the reluctance of the responsible governmental agencies such as the energy regulator NERSA (PBS, ENU, PRV). Second, the problems within creation of legitimacy were also identified as one of the main reasons for why the key actors and the public discourse focused on large-scale projects, which was one of the main problems within *guidance of the search* (ACD, ENU, NGO). Third, due to their resistance or reluctance, the key actors were also not willing to invest in resources that would have been necessary to support the technology such as technical infrastructure (e.g. grid monitoring systems, smart technology devices) or human resources in the electrical engineering departments (*resource mobilisation*) (ENU, PBS).

#### 5.3.2. Entrepreneurial activity – the ‘suffering’ function

Promblems witin *entrepreneurial activity* were affected by problems within three other functions. First, the considerable frustration prevailing among businesses was mainly a consequence of the problems within *market formation*, i.e. the lack of a clear legal framework, of market incentives, and of access to the grid (PRV, ACD, ENU). Second, both the frustration among businesses and the short-lived entrepreneurial landscape were at least to a certain degree also triggered by the lack of company competence and the fact that potential customers did not understand the costs and benefits of the technology, i.e. by problems within *knowledge development & diffusion* (PRV). One of the installers, for instance, reported the following: ‘High investment – you spend a lot of time with the customer with very little return – it is frustrating’. Third, businesses were also discouraged by the fact that key actors were focussing mainly on large-scale projects, one of the main problem within *guidance of the search* (PRV).

#### 5.3.3. Knowledge development & diffusion

The weak function *knowledge development & diffusion* was the only function that directly contributed to the problems within *creation of legitimacy*. The interviewees, including the municipal officials, clearly emphasised the lack of an appropriate implementation strategy, which held municipalities off from supporting PV technology (PBS, PRV, ENU). They argued that municipalities would only decrease their resistance, if they were able to maintain their revenue and connect the system to the grid at the same time. Furthermore, the interviewees highlighted the fact that key actors resisted supporting the technology simply because they did not understand how it was affecting them (PBS, PRV). Additionally, *knowledge development & diffusion* showed linkages to three other functions. The problems within the function affected *entrepreneurial activity* (link described above) and, themselves, were affected by problems within *guidance of the search* and *resource mobilisation*. More precisely, the development of implementation strategies was blocked by the lack of human resources and the focus of academia on large-scale technology (*guidance of the search*), as well as by the absence of necessary technical infrastructure (PRV, ENU).

## 6. Discussion

The findings of our study show that distributed PV technology suffered from a severe lack of legitimacy in South Africa. It was primarily the municipalities, which opposed large-scale implementation of the technology. Owing to the financial dependence of municipalities on the revenue stream from electricity sales, a flourishing distributed PV market segment was not in their interest, as this would probably have meant that they lose primarily those customers who pay the highest electricity tariffs (i.e. high-income households, commercial and industrial sites). The fact that municipalities used the bulk of their electricity sale profits as cross-subsidies in order to provide poor households with cheaper or even free electricity hardened their position.

Our analysis, however, also reveals that the technology's lack of legitimacy was only one of multiple problems, all of which were interconnected. On a conceptual level, this means that the identified problems not only hampered the performance of their associated system function, but also triggered or at least added to problems within other functions. For instance, the municipalities’ resistance not only constituted a problem within the function *creation of legitimacy*, but were also the reason for problems within the function *market formation* as the municipalities, inter alia, prevented access to the grid, which constituted a precondition for the large-scale implementation of distributed PV applications. The municipalities’ resistance in turn was reinforced by the lack of adequate implementation strategies, i.e. problems within the *knowledge development* function.

The visualisation of such interaction patterns allowed us to analyse the different roles that each of the system functions played in hampering the overall performance of the innovation system. In other words, analysing patterns of interaction at the level of systemic problems allowed us to improve our understanding of why and how system functions might interrelate. This makes our study distinct to previous research on systemic problems, which considered the interaction between system functions less explicitly (Turner et al. 2016; Wesseling and Van der Vooren 2016). In fact, we looked at the interplay between systemic problems in order to analyse how the functions interrelate. This procedural step is likely to contribute to a better understanding of the dynamics of cumulative causation across system functions and thus to earlier work in this field (e.g. Suurs and Hekkert 2009; Suurs et al. 2010). Indeed, the findings of our analysis indicate the existence of two vicious cycles; i.e. systemic lock-in situations, where mal-performing system functions reinforced each other (see Box 1).

Box 1. **Patterns of cumulative causation.**

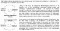

The practical advantages of understanding the specific roles and impacts of each of the functions is that it reveals those processes within the innovation system that require particular attention and thus allows policy and decision makers to develop effective intervention strategies. Furthermore, considering the functions’ relationship to the system structure indicates what part of the system needs to be tackled. For instance, while our analysis reveals that the inability to create legitimacy had the widest impact throughout the system, it also shows that strengthening the technology's legitimacy required to develop strategies that would (i) help municipalities to implement the technology in a reasonable way, (ii) involve the government and particularly Eskom more actively, and (iii) create networks that lobby for the technology. As another example, the analysis illustrates the interlinkages of the blocked entrepreneurial spirit with other functional weaknesses and their corresponding structural deficiencies and indicates, for instance, that for promoting entrepreneurship, developing a clear regulatory framework would be indispensable.

Our analytical emphasis on the Western Cape Province raises the question of how valid the findings are for the rest of the country. The fact that the country's electricity system is highly centralised and the underlying structure in terms of the key actors and the institutional environment is very similar among the provinces indicates that the identified problem structures were also applicable to the other provinces. However, considering our interview sample, the generalizability of our findings to the whole country is difficult to prove. Another limitation that goes along with the spatial scope of our analysis relates to the limited attention that we paid to the relevant national actors such as the Department of Energy (DoE) or the state-owned energy company Eskom. Even though these actors seemed to have taken a rather passive attitude from the provincial perspective, it cannot be ruled out that certain departments, initiatives or individuals within the large and diverse structures of these organisations deviated from the overall organisational attitude and showed a more proactive stance. It remains an interesting endeavour for future research to depict a more differentiated view of these organisations and of their role for the diffusion of distributed types of PV technology.

## 7. Conclusions

Our findings show that, in the Western Cape Province of South Africa, the diffusion of distributed PV technology was hampered by a broad range of interlinked systemic problems. The fact that the identified problems strongly interrelated also implied certain dynamics among their corresponding system functions. Moreover, by revealing these dynamics, we could show that each of the mal-performing functions played differed roles within the innovation system. Although the findings indicate the high level of complexity that characterise innovation problems, they also indicate that these problems may be addressed successfully; the analysis of interaction patters, both among systemic problems, and among system functions, not only sheds light on how and where an innovation system's performance is weak, but also allows decision- and policy-makers to develop strategies that consider the interplay of the relevant problems, instead of solving them in isolation. For future research, it would be highly interesting to compare our findings with the current situation of distributed PV technology in South Africa in order to take a longitudinal perspective on the associated problem structures. This would allow TIS scholars and practitioners to gain a deeper understanding of the dynamics among systemic problems (and associated system functions) over time.

## Notes

1 For detailed explanation of the notion of cumulative causation see Myrdal (1957).

2 There are two slightly different lists of system functions in the TIS literature. The main difference between them is that the list suggested by Bergek et al. 2008 also includes a function called development of positive externalities, which describes systemic benefits that occur due to the efforts of individual actors. However, such benefits are the result of the interplay of the other functions and are therefore included in the analysis already.

3 However, scholars have highlighted the high information value of interviews before (e.g. Bergek et al. 2008) and in recent years an increasing number of scholars have used interviews as the main source of information (e.g. Blum, Bening, and Schmidt 2015; Wesseling and Van der Vooren 2016).

4 Eskom owns the majority of the trans-regional transmission lines and operates fourteen large-scale coal-fired power plants that produce more than 90 % of electricity utilised in South Africa (Eskom 2016).

5 In the Western Cape Province (as well as in the rest of the country), the municipalities owe the majority of the distribution grids and are also in charge of who is getting connected to the grid.
